# First case report of inducible heart block in Lyme disease and an update of Lyme carditis

**DOI:** 10.1186/s12879-019-4025-0

**Published:** 2019-05-16

**Authors:** Don Walter Kannangara, Sindhu Sidra, Patel Pritiben

**Affiliations:** St Luke’s University Health Network, Warren Campus, 185 Roseberry Street, Phillipsburg, NJ 08865 USA

**Keywords:** Lyme disease, Lyme Carditis, Heart block, Tick(s), *Borrelia burgdorferi*

## Abstract

**Background:**

Lyme disease (LD), is the most common vector-borne illness in the US and Europe, with predominantly cutaneous, articular, cardiac and neuro-psychiatric manifestations. LD affects all layers of the heart and every part of the conducting system. Carditis is a less common manifestation of LD. Heart block (HB) as the initial and sole manifestation of LD is rare. Inducible HB has never been reported in LD. We report a case of heart block (HB) inducible with exercise and reversible with rest.

**Case presentation:**

A 37-year-old male presented to the emergency department after experiencing two episodes of syncope while at work. He presented, with a heart rate of 57 bpm, and the ECG showed sinus bradycardia with first degree AV block. The PR interval was 480 ms (NL 120–200 ms). Physical exam was unremarkable. The cardiologist’s initial impression was vaso-vagal attack. He developed high degree AV block during a stress test for the initial work up, which resolved on cessation of exercise. A similar episode while walking in the hallway, resolved at rest. The high degree AV block appeared inducible with exercise and reversible with rest. His Lyme serology was strongly positive. He was treated with ceftriaxone and doxycycline. After completing treatment, the patient had a normal ECG and returned to work without limitations, doing manual labor.

**Conclusions:**

Manifestations of Lyme carditis (LC) vary from asymptomatic and symptomatic electrocardiographic changes and heart block (HB) reversible with treatment, to sudden death. HB as the sole and initial presentation of LC is rare. There have been no reports of inducible HB in LD. Here we present a case of inducible and reversible high degree HB in a case of LC and an update of literature. Exercise and stress testing should be avoided in suspected cases of LC until resolution of carditis. Lyme carditis should be suspected in individuals with cardiac manifestations in an endemic area, particularly in the younger patients with no other etiology evident.

## Background

LD is caused by members of the *Borrelia burgdorferi* sensu lato complex transmitted by *Ixodes scapularis* and *I pacificus* in the U.S*.* The estimated annual incidence in the U.S. is 300,000. 95% of reported cases come from 12 Northeastern and 2 North Midwestern states [1]. LC is a rarer manifestation of LD [2]. LC may present with or without other manifestations of LD and could involve all layers of the heart: myocardium [3–7], pericardium [8, 9], endocardium [10–13], or pancarditis [4, 14, 15]. Myocarditis could be focal [6, 16] or diffuse [14]. Any part of the conducting system could be affected, and conduction blocks of varying degrees (Table 1) are the most common. The incidence of LC varies from 0.2–10% in different reports [2, 17–20]. The actual incidence may be higher. A pediatric study of patients with Lyme disease found ECG changes in 29% [21]. LC could result in acute heart failure [22, 23], cardiogenic shock [23, 24], cardiac arrest [25–27], or sudden cardiac death [15, 28, 29]. We report a case of exercise induced progression of first-degree heart block to high degree AV block, with complete resolution on cessation of exercise, which could be reproduced. The patient made a complete recovery after treatment with intravenous ceftriaxone and doxycycline.

**Table 1:** ECG changes reported in Lyme carditis

First degree heart block [16, 17, 30–33]	Steere et al. (1980,1984), Naik et al. 2008, van der Linde (1991), Afari (2016), Tumminello (2017)
Wenckebach phenomenon [17, 34–38] (Mobitz type I)	Steere et al. (1984), Shah and Kanzaria (2012), Dobbs and Mugmon (2013), Lee and Sigla (2016), Bennett et al. (2016), Muhammad and Simmonelli (2018)
Mobitz type II [34, 38, 39]	Shah and Kanzaria (2012), Muhammad and Simonelli (2018), Kashou et al. (2018)
Complete Heart block / High degree AV block [17, 25, 30, 32, 34, 40–48]	Steere et al. (1980,1984), van der Linde (1990), Greenberg et al. (1997), Kline (2007) Bacino et al. (2011), Shah and Kanzaria 2012, Wenger et al. (2012), Dobbs and Mugman (2013), Jensen et al. (2014), Shah et al. (2015), Afari (2016), Timmer (2016), Afari (2016), Lee and Singla (2016), Chaudhry et al. (2017), Patel (2017)
Bundle branch block [26, 44, 49]	Khalil et al. (2015), Wenger et al. (2012), Cunha et al. (2017)
Sinus arrest / Sinus pauses [22, 50, 51]	Franck and Wollschläger (2003), Koene et al. (2012), Oktay et al. (2015)
Supraventricular tachycardia [6]	Konopka et al. (2013)
Atrial fibrillation [16, 44]	Naik et al. 2008, Wenger et al. (2012)
Junctional tachycardia [27, 52, 53]	Tanksley and Playe (2005), Frank et al. (2011), Cunningham et al. (2016)
Fascicular tachycardia [26, 41]	Greenberg et al. (1997), Khalil et al. (2015)
Ventricular tachycardia [22, 25, 54]	Vlay et al. (1991), Koene et al. (2012), Jensen et al. (2014)
Ventricular flutter [22]	Koene et al. (2012)
Bradycardia [16, 30, 37, 38, 42, 55]	Steere et al. 1980, Kline (2007), Naik et al. 2008, Abraham et al. (2010), Bennett et al. (2016) Muhammad and Simonelli (2018)
Narrow QRS escape rhythm [34]	Shah and Kanzaria (2012)
Prolonged QT [56, 57]	Seslar et al., (2006), Welsh et al. (2012)
ST depression / T inversion [30, 57]	Steere et al. (1980), Welsh et al. (2012)
ST elevation [58]	Michalski et al. (2017)
Asystole [26]	Khalil et al. (2015)
History of Wolf Parkinson White Syndrome In a case of sudden death due to LC [14]	CDC (2013)

## Case presentation

**Fig. 1:**
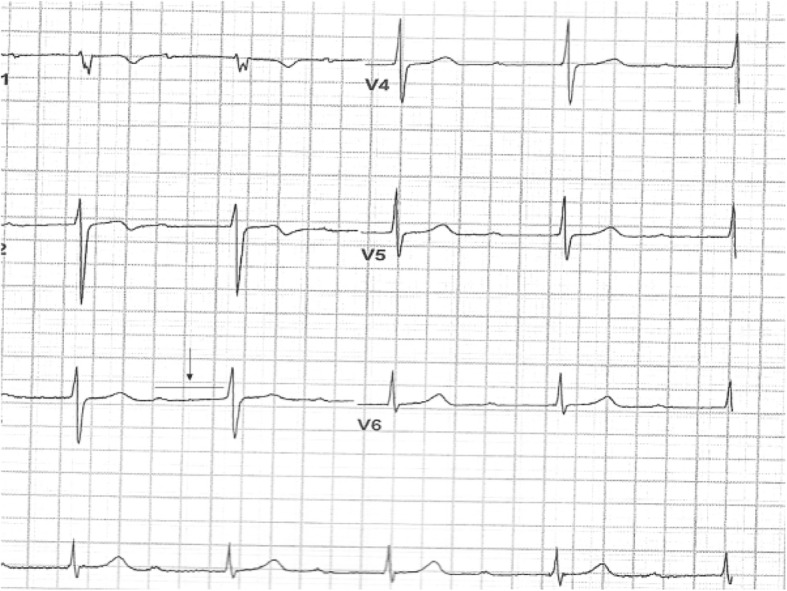
Admission ECG showing first degree heart block (PR = 480 ms)

**Fig. 2:**
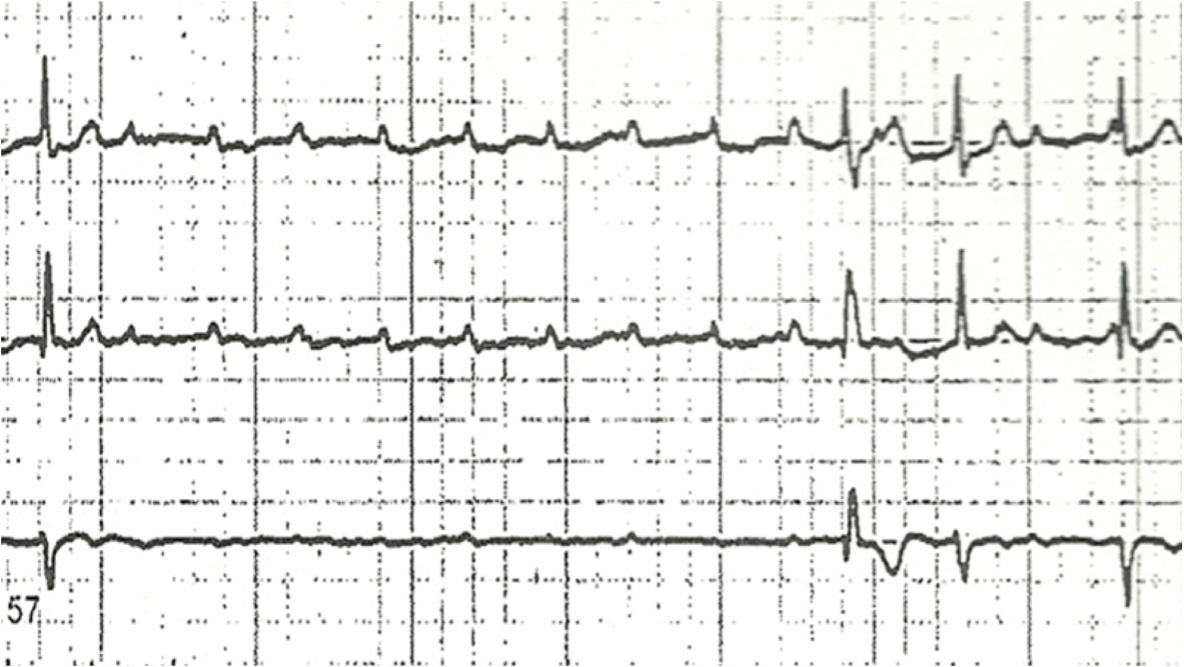
High degree A-V block during exercise

A 37-year-old male with past medical history significant for asthma, anxiety, and former tobacco use, presented to our emergency department after experiencing two episodes of syncope while at work. He was employed outdoors in a heavy manual labor industry. He and his co-workers have been frequently bitten by ticks at work in the past. Initial vital signs on admission were significant for bradycardia, with a heart rate of 57 bpm, and the ECG showed sinus bradycardia with first degree AV block, with a PR interval of 480 ms (NL 120–200 ms) (Fig. 1). Physical exam was unremarkable, except for hypopigmentation of fingers. Serum ALT level was elevated 115 (NL 12–78). Other labs on admission were all within normal including serum troponin. Further workup included a normal CT scan of head, a vascular study of the carotid vessels that showed minor right sided carotid stenosis of < 50%, and an echocardiogram that was unremarkable,except for mildly increased LV wall thickness with an EF of 60%. An exercise stress test done by the cardiologist, was terminated early. The patient developed dyspnea, and his ECG demonstrated progression of first-degree AV block to high degree AV block (Fig. 2). Once back at rest, the patient’s high degree AV block reverted to first degree AV block. He had a similar episode while walking in the hallway wearing a Holter monitor, on day 5, also reversible with rest. He was transferred to the critical care unit for close monitoring and treated with ceftriaxone 2G iv once daily and doxycycline 100 mg orally twice daily. The first-degree AV block improved with a gradual decrease in the PR interval (Table 2). His Lyme serology (Western Blot) was strongly positive (Table 3).

**Table 2:** PR interval decrease with treatment

DAY	1	4	5	6	7	16
PR Interval	480	416	440	316	284	178

**Table 3:** Lyme serology results

Lyme AB IGG	2.3 (0.00–0.79)
Lyme AB IGM	15.67 (0.00–0.79
Lyme 18 kD IgG	Present
Lyme 23 kD IgG	Present
Lyme 30 kD IgG	Present
Lyme 39 kD IgG	Present
Lyme 41 kG IgG	Present
Lyme 45 kG IgG	Present
Lyme 58 kG igG	Present
Lyme 23 kD IgM	Present
Lyme 39 kD IgM	Present
Lyme 41 kD IgM	Present

**Fig. 3:**
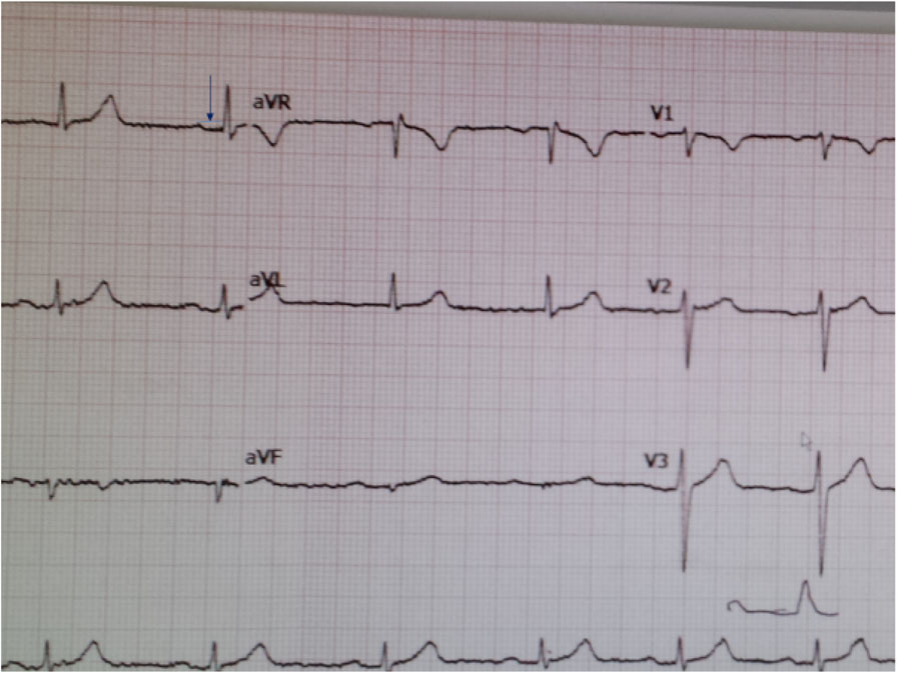
ECG after treatment showing normal PR interval (PR = 178 ms)

The heart block significantly improved to 270 ms by day 7 of treatment. He was discharged and continued outpatient IV Ceftriaxone for 3 weeks. After completing treatment, the patient had a normal ECG with PR interval of 178 (Fig. 3) on day 16 and an uneventful exercise stress test. He returned towork without limitations, doing manual labor. He has been symptom free for 2 years. Now he uses tick-repellents at work.

## Discussion and conclusions

Cardiac involvement has been reported with *B.burgdorferi* ss (US cases), *B garinii, B afzelli* [12] and *B bissettii* [10]. Patients with CHB (Complete heart block) due to LC may have erythema migrans [32, 36, 37, 47, 48, 58] or other manifestations such as joint involvement [8]. Basic investigations include the history of possible tick exposure, laboratory testing, a 12-channel electrocardiogram, 24-h Holter monitor, chest x-ray, and echocardiography [2]. The two-tier antibody-based test recommended by the CDC is highly specific but has poor sensitivity [59]. Supplementary studies that have been used in the diagnosis include echocardiography [7, 57, 60], gadolinium enhanced cardiac MRI [16, 27], endo-myocardial biopsy [6, 7, 22, 61, 62] with special staining [3, 4, 8, 40], culture [3] or electron microscopy [7, 61–63], Ga67 Scan [64–66], and Indium 111 labelled anti-myosin antibody scintigraphy [4]. Histology of affected cardiac tissue shows infiltration predominantly with lymphocytes and plasma cells [15, 22, 29, 40].

Progression of first-degree heart block to second degree and then to complete heart block without treatment has been reported. Regression of complete heart block to second degree, then first degree followed by complete resolution with treatment is also known [32]. Inducible heart block in Lyme disease has not been reported. We present the first report of inducible heart block in a patient whose initial and sole manifestation of LD was HB. First-degree heart block worsened to high degree AV block on exercise, which was reversible with rest and reproducible during the hospitalization.

As illustrated in this case, exercise and stress testing should not be carried out in LD patients until complete recovery from heart block. HB secondary to Lyme disease rarely requires a permanent pacemaker. However, patients with a PR interval greater than 300 ms should be monitored in an intensive care setting, as they may rapidly progress to complete HB [30]. A temporary pacer is sometimes required; however, most patients respond to treatment within two to three weeks. The antibiotics used in LC include amoxicillin [12], ceftriaxone [8, 9, 32, 36, 38, 41, 53], doxycycline [49] and ceftriaxone and doxycycline [13, 37]. Duration of treatment varied from 2 weeks to one month.

LC may present with HB as the sole [53] or initial presentation [45, 66] with or without other manifestations of LD [30, 32, 36, 37, 48] or with multiple electrocardiographic (Table 1) and clinical presentations. First degree HB is the most common manifestation, which could rapidly progress to CHB [32]. It is important to keep a high degree of suspicion for Lyme disease in endemic areas in patients with cardiac symptoms with or without other manifestations of LD, particularly younger individuals with no other etiology evident. Some patients with complete heart block may need a temporary pacemaker [26, 39, 47, 55, 66, 67]. The majority of AV blocks in LC are reversible with antibiotic treatment. Exercise is contraindicated until resolution of LC.

## Abbreviations

ALT: Alanine aminotransferase
AV: Atrioventricular
BPM: Beats per minute
CDC: Center for disease control
CHB: Complete heart block
CT: Computed tomography
ECG: Electrocardiogram
EF: Ejection fraction
HB: Heart block
IGG: Immunoglobulin G
IGM: Immunoglobulin M
IV: Intravenous
LC: Lyme carditis
LD: Lyme Disease
LV: Left ventricle
MRI: Magnetic resonance imaging
MS: Milli seconds
NL: Normal limits
